# Treatment Patterns, Outcomes, and Costs Associated With Localized Upper Tract Urothelial Carcinoma

**DOI:** 10.1093/jncics/pkab085

**Published:** 2021-10-01

**Authors:** Katherine E Fero, Yong Shan, Patrick M Lec, Vidit Sharma, Aditya Srinivasan, Giri Movva, Jacques Baillargeon, Karim Chamie, Stephen B Williams

**Affiliations:** 1 Department of Urology, University of California Los Angeles, Los Angeles, CA, USA; 2 Department of Surgery, Division of Urology, The University of Texas Medical Branch, Galveston, TX, USA; 3 Department of Medicine, Division of Epidemiology, Sealy Center on Aging, The University of Texas Medical Branch at Galveston, Galveston, TX, USA

## Abstract

**Background:**

Upper tract urothelial carcinoma (UTUC) is a heterogeneous disease that presents a clinical management challenge for the urologic surgeon. We assessed treatment patterns, costs, and survival outcomes among patients with nonmetastatic UTUC.

**Methods:**

We identified 4114 patients diagnosed with nonmetastatic UTUC from 2004 to 2013 in the Survival Epidemiology, and End Results–Medicare population-based database. Patients were stratified into low- or high-risk disease groups. Median total costs from 30 days prior to diagnosis through 365 days after diagnosis were compared between groups. Overall and cancer-specific survival were evaluated using Cox proportional hazards regression. All statistical tests were 2-sided.

**Results:**

After risk stratification, 1027 (24.9%) and 3087 (75.0%) patients were classified into low- vs high-risk UTUC groups. Most patients underwent at least 1 surgical intervention (95.1%); 68.4% underwent at least 1 endoscopic intervention. Patients diagnosed with high- vs low-risk UTUC were more likely to undergo nephroureterectomy (83.6% vs 72.0%; *P *<* *.001); few patients with low-risk disease were exclusively managed endoscopically (16.9%). At 365 days after diagnosis, costs of care for high- vs low-risk UTUC were statistically significantly higher ($108 520 vs $91 233; median difference $16 704, 95% confidence interval [CI] = $11 619 to $21 778; *P *<* *.001). Those with high-risk UTUC had worse cancer-specific and overall survival compared with patients with low-risk UTUC (cancer-specific survival hazard ratio [HR] = 4.14, 95% CI = 3.19 to 5.37; overall survival HR = 1.78, 95% CI = 1.62 to 1.96).

**Conclusions:**

UTUC continues to be managed primarily with nephroureterectomy, regardless of risk stratification, and patients with high-risk UTUC have worse overall and cancer-specific survival. Substantial costs are associated with management of low- and high-risk UTUC, with the latter being more costly up to 1 year from diagnosis.

Urothelial cell carcinoma of the upper tract (UTUC) is a heterogeneous disease with increased mortality with more advanced stage (1). Epidemiologic data suggest the incidence of UTUC is rising, whereas survival remains relatively unchanged (2-4). Management of patients with UTUC is highly variable and depends on a number of factors including presence of a contralateral kidney, baseline renal function, tumor location (renal pelvis vs ureter), size, grade, clinical stage, and focality (5). Preoperative risk stratification has helped guide decision making regarding surgical management: for patients with high-risk disease (multifocal tumors, those >2 cm, high-grade cytology, muscle invasive on biopsy, or suggestion of infiltration on imaging), the gold standard surgical management remains radical nephroureterectomy, bladder cuff excision, and lymphadenectomy (if suspicion of or confirmed muscle invasion) (6). However, kidney-sparing approaches, such as distal and segmental ureterectomy or ureteroscopic ablation, have been shown to have similar oncologic outcomes to extirpative surgery in carefully selected patients (6-9).

The natural trade-off of renal preservation during treatment of UTUC is the increased burden of surveillance via imaging and cystoscopy (10). Given the complexity of UTUC management to include diagnostic interventions, ablative or extirpative surgery, perioperative and longer-term sequelae of these interventions, and surveillance and follow-up, caring for UTUC patients is likely a costly endeavor. However, only 1 study on cost after UTUC diagnosis, reporting exclusively on low-risk patients at a single institution, has been published (11).

We aimed to characterize the treatment patterns, survival outcomes, and costs for patients with nonmetastatic low- and high-risk UTUC. We hypothesized that overall renal preservation strategies remain infrequently used, caring for patients with high-risk disease is associated with increased costs, and there is increased mortality among those with high-risk disease.

## Methods

### Data Source

A population-based retrospective cohort study was conducted using the Surveillance, Epidemiology, and End Results (SEER) and Medicare–linked database. The SEER database contains demographic and clinical data pooled from individual population-based cancer registries covering approximately 30% of the US population. Medicare provides federally funded health insurance for individuals aged 65 years and older in the United States; a 5% random sample is included in the Medicare portion of the linked dataset. The SEER-Medicare linkage includes claims data for patients within SEER and spans a time period prior to diagnosis through death (12). The study was reviewed by the institutional review board (IRB) at the University of Texas Medical Branch; patient informed consent was waived by the IRB. We performed data analysis from July 1, 2020, to March 1, 2021.

### Ascertainment of Study Cohort

**Figure 1.**
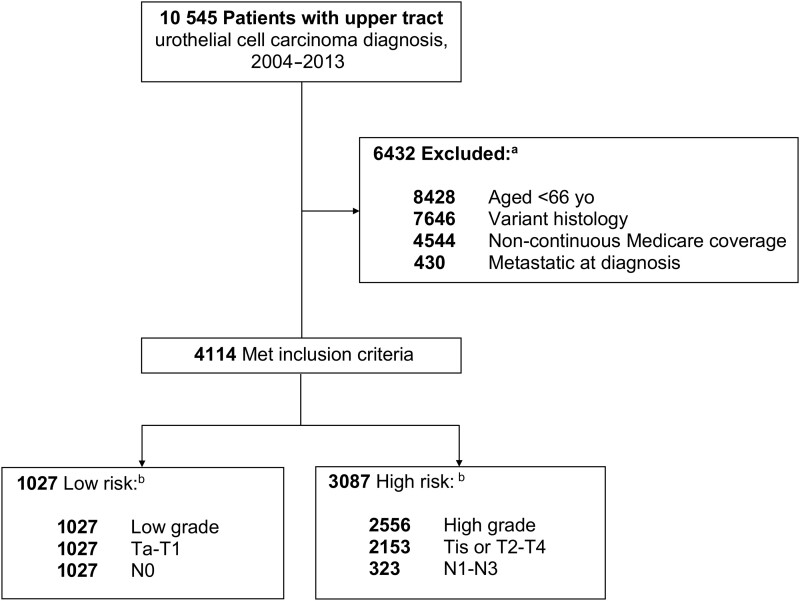
Patient selection process. ^a^Some patients met more than 1 exclusion criterion. ^b^Some patients met more than 1 risk classification criterion.

In the SEER-Medicare database 2015 linkage, we identified 4114 patients diagnosed with urothelial cell carcinoma (histology codes 8130, 8120, 8131) of the ureter (site code: C669) or renal pelvis (site code: C659) during the study period of 2004-2013 to allow for at least 1 year of follow-up after diagnosis. We restricted our analysis to those aged 66 years and older at diagnosis to allow a full year of prediagnosis claims data from which to calculate a comorbidity score. Patients with incomplete coverage data were excluded, as were those without continuous Medicare coverage (parts A and B) or with any part C coverage (health maintenance organization enrollment). We excluded patients with metastatic cancer at the time of diagnosis. Figure  1 depicts the comprehensive schema of the cohort selection.

### Study Covariates

Patient age and stage at diagnosis, year of diagnosis, sex, race, marital status, histologic grade, and tumor location were extracted from SEER. County-level median annual household income was acquired through linkage to the Area Health Resource Files data and then divided into quartiles. Comorbidities were assessed from the Medicare claims data using the Klabunde modification of the Charlson comorbidity index in the year prior to UTUC cancer diagnosis (13).

Treatment variables were identified from Medicare claims data. Type of surgery was categorized as endoscopic intervention or nephroureterectomy; this was captured via International Classification of Disease–9 and Current Procedural Terminology codes from 30 days prior to diagnosis through 1 year after diagnosis (see Supplementary Table 1, available online). An additional variable of “definitive” surgical intervention was defined for patients who underwent multiple surgeries (ie, if a patient underwent a ureteroscopy, followed by a nephroureterectomy, the nephroureterectomy was considered the definitive procedure). The total number of surgical procedures undergone by an individual patient was summed.

To determine treatment costs, we summed all Medicare health-care expenditures from inpatient, outpatient, and physician services from 30 days prior to diagnosis to 90 days, 180 days, and 365 days after diagnosis. All costs were inflated to 2020 US dollars using previously established methods summing Medicare reimbursements, coinsurance reimbursements, and patient liability (14,15).

### Patient Groups

Patients were categorized as either low- or high-risk based on stratification adapted from the European Association of Urology guidelines; this stratification was modified based on available characteristics in the SEER database to include grade and pathologic stage (6). Thus, in this study, low risk was defined as low grade on histology and American Joint Committee on Cancer (AJCC) stage Ta or T1 and N0. High-risk patients were all others: high-grade histology or AJCC tumor stage of 2 or more or nodal stage of 1 or more. Tumor size was not included in our adapted risk stratification because of a large proportion of missing data (21%).

### Statistical Analysis

Descriptive statistics were used to compare groups of patients by risk classification. χ^2^ tests were used to compare differences between proportions of patients in each group. Median costs were compared between groups of patients; as this cost data are not normally distributed, we used Hodges-Lehmann nonparametric estimator and 2-sample Wilcoxon test to evaluate the difference between groups. Kaplan-Meier curves were constructed to assess overall and cancer-specific survival, stratified by risk group; patients were included from time of diagnosis until death or last available follow-up (December 31, 2014). A multivariable Cox proportional hazards regression model was used to perform adjusted survival analysis; variables included in the model were defined a priori and consisted of available sociodemographic and clinical covariates. The proportional hazard assumption in the Cox models was tested by checking that the logarithm of the baseline cumulative hazard rates and the Schoenfield residuals were proportional to follow-up time. A *P* value of less than .05 was considered statistically significant for all analyses. All statistical analyses were conducted using SAS 9.4 (SAS Institute Inc, Cary, NC).

## Results

After risk stratification of the 4114 patients in the study cohort, 1027 (24.9%) and 3087 (75.0%) patients were classified as low- vs high-risk UTUC (Table  1). Women made up a higher proportion of patients with high-risk disease vs low-risk disease (47.8% vs 42.6%; *P *=* *.003). Older patients were overrepresented in the high-risk group (aged older than 80 years: 40.7% vs 36.0%; *P *=* *.003). Tumor location also differed statistically significantly between groups, with renal pelvis tumors comprising a larger proportion among those with high- vs low-risk disease (61.5% vs 50.1%; *P *<* *.001), respectively.

**Table 1.** Patient demographics according to risk stratification^a^

Variable	Total cohort, No. (%)	Low risk^b^, No. (%)	High risk^b^, No. (%)	*P* ^c^
Sex				.003
Male	2200 (53.5)	590 (57.4)	1610 (52.2)	
Female	1914 (46.5)	437 (42.6)	1477 (47.8)	
Age at diagnosis, y				.003
66-70	612 (14.9)	184 (17.9)	428 (13.9)	
71-75	920 (22.4)	243 (23.7)	677 (21.9)	
76-80	956 (23.2)	230 (22.4)	726 (23.5)	
>80	1626 (39.5)	370 (36.0)	1256 (40.7)	
Race				.13
Black	128 (3.1)	29 (2.8)	99 (3.2)	
Hispanic	56 (1.4)	11 (1.1)	45 (1.5)	
White	3702 (90.0)	943 (91.8)	2759 (89.3)	
Other	228 (5.5)	44 (4.3)	184 (6.0)	
Marital status				.80
Single	497 (12.1)	121 (11.8)	376 (12.2)	
Married	2331 (56.6)	591 (57.5)	1740 (56.4)	
Unknown	1286 (31.3)	315 (30.7)	971 (31.5)	
Census region				.01
West	1615 (39.3)	364 (35.4)	1251 (40.5)	
Midwest	929 (22.6)	264 (25.7)	665 (21.5)	
South	487 (11.8)	125 (12.2)	362 (11.7)	
Northeast	1083 (26.3)	274 (26.7)	809 (26.2)	
Median income				.50
Bottom quartile	1095 (26.6)	286 (27.8)	809 (26.4)	
Second quartile	1033 (25.1)	263 (25.6)	770 (24.9)	
Third quartile	1003 (24.4)	234 (22.8)	769 (24.9)	
Fourth quartile	983 (23.9)	244 (23.8)	739 (23.9)	
No. of comorbidities				.12
0	1817 (44.2)	429 (41.7)	1388 (45.0)	
1	1023 (24.9)	268 (26.1)	755 (24.4)	
2	597 (14.5)	167 (16.3)	430 (13.9)	
≥3	677 (16.5)	163 (15.9)	514 (16.7)	
Year of diagnosis				.55
2004	458 (11.1)	115 (11.2)	343 (11.1)	
2005	424 (10.3)	115 (11.2)	309 (10.0)	
2006	405 (9.8)	82 (8.0)	323 (10.5)	
2007	415 (10.1)	107 (10.4)	308 (10.0)	
2008	455 (11.1)	117 (11.4)	338 (10.9)	
2009	446 (10.8)	119 (11.6)	327 (10.6)	
2010	382 (9.3)	89 (8.7)	293 (9.5)	
2011	389 (9.5)	100 (9.7)	289 (9.4)	
2012	364 (8.8)	87 (8.5)	277 (9.0)	
2013	376 (9.1)	96 (9.3)	280 (9.1)	
Grade				<.001
Low	1318 (32.0)	1027 (100)	291 (9.4)	
High	2556 (62.2)	0 (0)	2556 (82.8)	
Unknown	240 (5.8)	0 (0)	240 (7.8)	
AJCC T stage				<.001
Ta	998 (24.3)	680 (66.2)	318 (10.3)	
T1	921 (22.4)	347 (33.8)	574 (18.6)	
T2	557 (13.5)	0 (0)	557 (18.0)	
T3	1136 (27.6)	0 (0)	1136 (36.8)	
T4	266 (6.5)	0 (0)	266 (8.6)	
Tis	194 (4.7)	0 (0)	194 (6.3)	
Unknown	42 (1.0)	0 (0)	42 (1.4)	
AJCC N stage				<.001
Nx	57 (1.4)	—	57 (1.8)	
N0	3734 (90.8)	1027 (100)	2707 (87.7)	
N1	189 (4.6)	0 (0)	189 (6.1)	
N2	128 (3.1)	0 (0)	128 (4.1)	
N3	—	—	—	
Unknown	—	—	—	

a Cell counts less than 11 are designated with “—” and cannot be reported as mandated by Surveillance, Epidemiology and End Results–Medicare data use agreement for privacy concerns. AJCC = American Joint Committee on Cancer.

b Patients were stratified into low- and high-risk disease groups based on criteria adapted from the European Association of Urology guidelines (6).

c *P* values were calculated with a 2-sided χ^2^ test.

**Figure 2.**
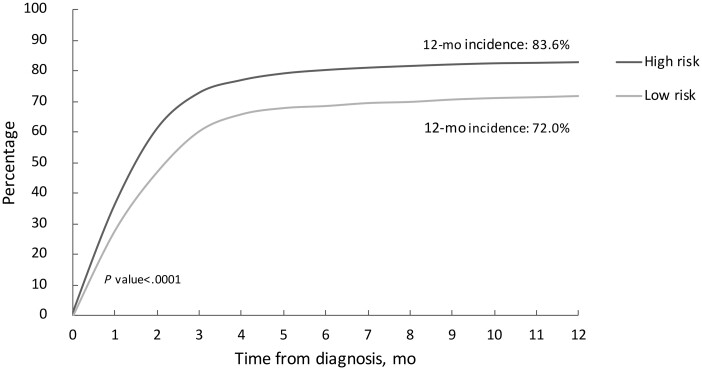
Cumulative incidence of nephroureterectomy according to risk stratification. The *P* value was calculated with a 2-sided χ^2^ test.

Comprehensive treatment details according to risk strata are included in Table  2. The majority of patients underwent at least 1 diagnostic and/or therapeutic surgical intervention (95.1%). Of the total cohort, 71% (2920 of 4114) of patients underwent an endoscopic intervention. More low-risk patients underwent any endoscopy compared with high-risk patients (78.9% vs 68.4%; *P *<* *.001). Iterative endoscopic interventions were more commonly performed among patients with low-risk disease: 16.6% of these patients underwent 2 endoscopic procedures in the year after diagnosis and 16.7% underwent 3 or more. A minority of patients were managed exclusively endoscopically, although this was more common among those with low-risk disease, as compared with high risk (16.9% vs 6.7%, respectively; *P *<* *.001). Similarly, it was more common for patients with ureteral tumors, regardless of risk strata, to be managed exclusively endoscopically than for those with tumors of the renal pelvis (13.3% vs 6.4%, respectively; *P *<* *.001). Of the patients with high-risk disease, 82% underwent nephroureterectomy within a year of diagnosis vs 71.8% of patients with low-risk disease (Figure  2). Patients with tumors in the renal pelvis were more likely to undergo nephroureterectomy within a year of diagnosis than those with ureteral tumors, regardless of risk strata (83.6% vs 72.0%, respectively; *P *<* *.001). Among patients who underwent both endoscopic interventions and nephroureterectomy, the median time from endoscopy to nephroureterectomy was 36 days (interquartile range = 20-57) among patients with low-risk disease and 30 days (interquartile range = 16-47) among those in the high-risk group. The rate of lymph node dissection was low overall, although more commonly performed among patients with high-risk disease compared with low-risk disease (22.1% vs 9.4%, respectively; *P *<* *.001). Systemic therapy prior to surgical intervention was rare; 12 (0.3%) patients underwent neoadjuvant chemotherapy. Adjuvant chemotherapy was administered to more patients in the high-risk group (17.6%) compared with the low-risk group (2.4%) (*P *<* *.001).

**Table 2.** Treatments according to risk stratification

Variable	Total cohort, No. (%)	Low risk, No. (%)	High risk, No. (%)	*P* ^a^
Adjuvant systemic chemotherapy				<.001
Yes	567 (13.8)	25 (2.4)	542 (17.6)	
No	3547 (86.2)	1002 (97.6)	2545 (82.4)	
Any endoscopic intervention				<.001
Yes	2920 (71.0)	810 (78.9)	2110 (68.4)	
No	1194 (29.0)	217 (21.1)	977 (31.6)	
No. of endoscopic interventions				<.001
0	1194 (29.0)	217 (21.1)	977 (31.6)	
1	1983 (48.2)	468 (45.6)	1515 (49.1)	
2	584 (14.2)	170 (16.6)	414 (13.4)	
≥3	353 (8.6)	172 (16.7)	181 (5.9)	
Segmental ureterectomy				.02
Yes	443 (10.8)	130 (12.7)	313 (10.1)	
No	3671 (89.2)	897 (87.3)	2774 (89.9)	
Radical nephroureterectomy				<.001
Yes	3321 (80.7)	739 (72.0)	2582 (83.6)	
No	793 (19.3)	288 (28.0)	505 (16.4)	
Definitive surgical intervention				<.001
Endoscopic only	381 (9.3)	174 (16.9)	207 (6.7)	
Laparoscopic/open surgery	3532 (85.8)	806 (78.5)	2726 (88.3)	
None	201 (4.9)	47 (4.6)	154 (5.0)	
Lymph node dissection				<.001
Yes	780 (19.0)	97 (9.4)	683 (22.1)	
No/unknown^b^	3334 (81.0)	930 (90.6)	2404 (77.9)	
Lymph node status				<.001
Positive	233 (5.7)	0 (0)	233 (7.5)	
Negative	547 (13.3)	97 (9.4)	450 (14.6)	
Unknown	3334 (81.0)	930 (90.6)	2404 (77.9)	

a *P* values were calculated with a 2-sided χ^2^ test.

b Unknown cell counts for lymph node dissection were all less than 11 and were collapsed into “No” category as they cannot be reported per Surveillance, Epidemiology and End Results–Medicare data use agreement for privacy concerns.

The median cost of all care in the total cohort at 90 days, 180 days, and 365 days after diagnosis was $57 945, $75 502, and $103 746, respectively. By 180 days after diagnosis, median costs of care for high-risk patients were statistically significantly higher than low-risk patients ($77 770 vs $69 249; median difference = $9811, 95% confidence interval [CI] = $6282 to $13 339; *P *<* *.001), and this differenced widened by 365 days after diagnosis ($108 520 vs $91 233; median difference = $16 704, 95% CI = $11 619 to $21 788; *P *<* *.001). As time from diagnosis lengthened, outpatient costs made up a larger proportion of total costs, as compared with inpatient costs (Table  3).

**Table 3.** Median inpatient and outpatient costs according to risk stratification

Costs	Total cohort, $	Low risk, $	High risk, $	*P* ^a^	High-low estimates (95% CI), $^b^
90-day costs					
Inpatient	17 216	14 891	17 844	<.001	−3318 (-4449 to -2187)
Outpatient	36 123	36 743	35 836	.51	490 (-1516 to 2496)
180-day costs					
Inpatient	19 711	17 534	20 419	<.001	−3701 (-4781 to -2621)
Outpatient	49 213	45 406	51 040	<.001	−4835 (-7616 to -2053)
365-day costs					
Inpatient	23 162	20 169	24 288	<.001	−4191 (-5432 to -2950)
Outpatient	70 651	63 810	73 456	<.001	−9621 (-13 635 to -5607)

a *P* values calculated with a 2-sample Wilcoxon test to evaluate the difference between groups. All statistical tests were 2-sided. CI = confidence interval.

b Difference between low- and high-risk groups (high-low estimate, 95% CI) in 2020 US dollars.

Costs were determined for the subset of patients who underwent any surgical intervention (n = 3913) according to risk stratification (Table  4). As expected, 90, 180, and 365 days postdiagnosis, median costs were statistically significantly higher with increased number and type of procedures performed but also according to high- vs low-risk disease. For example, high- vs low-risk patients had higher costs according to those who underwent a single endoscopic intervention ($77 929 vs $69 416; *P *=* *.21), nephroureterectomy ($89 613 vs $60 934; *P *<* *.001), or an endoscopic intervention with subsequent nephroureterectomy ($111 880 vs $83 847; *P *<* *.001). One-year costs for patients who underwent 2 endoscopic surgeries only were comparable with 1-year costs for patients who underwent either 1 or 2 endoscopic surgeries followed by nephroureterectomy, regardless of risk strata (2 endoscopies only: $104 508; 1 endoscopy with nephroureterectomy: $103 770; 2 endoscopies with nephroureterectomy: $126 895). However, 1-years costs differed according to risk stratification with high- vs low-risk patients having higher costs despite undergoing similar type(s) of procedures (ie, 2 endoscopies with nephroureterectomy: $131 067 vs $119 294; *P *=* *.13).

**Table 4.** Median costs according to surgical intervention (n = 3913) and risk stratification

Costs	Total cohort, $	Low risk, $	High risk, $	*P* ^a^	High-low estimate (95% CI), $^b^
Total 90-day costs					
1 endoscopy	49 862	49 126	49 862	.42	−5302 (−18 440 to 7837)
2 endoscopies	70 767	71 343	66 821	.95	2105 (−18 117 to 22 328)
NU only	44 933	37 685	47 775	<.001	−9826 (−13 956 to −5695)
1 endoscopy + NU	60 324	55 203	61 683	<.001	−6849 (−10 359 to −3339)
2 endoscopies + NU	76 186	80 219	74 293	.47	2928 (−4868 to 10 723)
Total 180-day costs					
1 endoscopy	56 470	55 433	58 300	.20	−12 853 (−30 849 to 5142)
2 endoscopies	85 093	85 093	85 674	.49	−8206 (−33 909 to 17 497)
NU only	58 729	41 786	63 747	<.001	−20 344 (−26 746 to −13 941)
1 endoscopy + NU	76 615	66 866	80 029	<.001	−14 385 (−18 982 to− 9788)
2 endoscopies + NU	97 918	97 644	98 295	.52	−3271 (−13 238 to 6697)
Total 365-day costs					
1 endoscopy	72 463	69 416	77 929	.21	−15 196 (−38 022 to 7630)
2 endoscopies	104 508	98 090	111 096	.55	−8600 (−41 096 to 23 897)
NU only	81 787	60 934	89 613	<.001	−27 879 (−37 644 to −18 114)
1 endoscopy + NU	103 770	83 847	111 880	<.001	−26 491 (−33 090 to −19 892)
2 endoscopies + NU	126 895	119 294	131 067	.13	−10 682 (−24 444 to 3081)

a *P* values calculated with a 2-sample Wilcoxon test to evaluate the difference between groups. All statistical tests were 2-sided. CI = confidence interval; NU = nephroureterectomy.

b Difference between low- and high-risk groups (high-low estimate, 95% CI) in 2020 US dollars.

**Figure 3.**
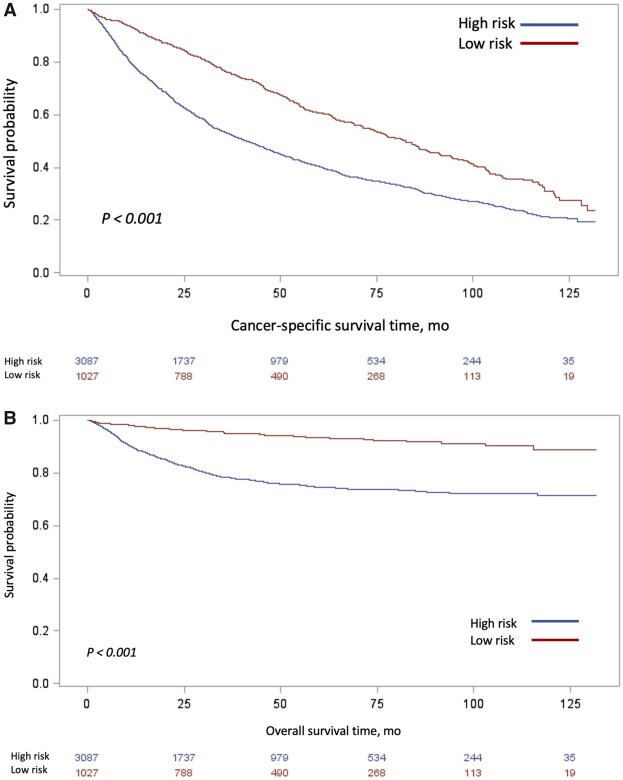
Unadjusted Kaplan-Meier curves of **(A)** overall survival and **(B)** cancer-specific survival, stratified by risk classification. Tables of the numbers of patients at risk in the high- and low-risk groups are below each graph.

Median follow-up was 74.8 months. Median overall survival was 82.4 months (95% CI = 75.0 to 88.1 months) among those in the low-risk group and 41.2 months (95% CI = 38.0 to 44.5 months) among those in the high-risk group (Figure  3). There were 2369 patient deaths in our cohort during the study period. In unadjusted analysis, patients with high-risk UTUC were at increased risk of cancer-specific mortality (HR = 4.1, 95% CI = 3.2 to 5.4) and death from any cause (HR = 1.8, 95% CI = 1.6 to 2.0) (Supplementary Figure 1, available online). After controlling for other clinical and demographic covariates, the increased risk of cancer-specific mortality and all-cause mortality persisted among those with high- vs low-risk disease ( cancer-specific survival: HR = 4.14, 95% CI = 3.19 to 5.37; overall survival: HR = 1.78, 95% CI = 1.62 to 1.96) (Supplementary Table 2, available online).

## Discussion

In this large, nationally representative retrospective cohort study of more than 4000 older adults with nonmetastatic UTUC, most patients underwent nephroureterectomy within a year of diagnosis. When compared with patients with low-risk disease, those with high-risk tumors had statistically significantly higher costs and shorter overall survival time.

Patients with high-risk disease comprised three-quarters of the study cohort; inclusion in the high-risk strata was predominantly driven by presence of high-grade disease. As recommended, most patients (82.8%) with high-risk tumors underwent radical nephroureterectomy (16). However, we also found that the majority of low-risk UTUC patients studied underwent radical nephroureterectomy within a year of diagnosis, despite a body of literature promoting a paradigm shift toward renal preservation strategies for select patients with low-risk disease (6,7,17-20). In fact, nearly 30% of all patients never underwent ureteroscopy. A similar proportion has been reported at the institutional level: in a study of 155 patients with UTUC, 28% proceeded to nephroureterectomy based on imaging characteristics and cytology alone (21). Importantly, the authors also reported that disease status was not more severe among patients who had a brief delay to nephroureterectomy because of ureteroscopy with biopsy and/or ablation. Furthermore, recently published data suggest that nearly 50% of patients had changes made to their treatment plans based on diagnostic ureteroscopy findings following computerized tomography urography (22). Taken as a whole, one may question if diagnostic ureteroscopy is underutilized.

Although, in our study, 80% of patients with low-risk disease underwent at least 1 endoscopic intervention, only 16.9% of these patients did not subsequently undergo nephroureterectomy. A minority (16.7%) of patients with low-risk disease underwent iterative endoscopic procedures in the year after diagnosis, suggesting attempt at management with renal preservation among these patients. Two prior studies of cancer registry data, during roughly the same time frame, also reported a low proportion of potentially eligible patients who received kidney-sparing surgeries (23,24). This underutilization of kidney-sparing surgery among patients who may be eligible for such therapy highlights an important opportunity for improvement in patient-centered care. A related recent development is the Food and Drug Administration approval of a novel formulation of intracavitary mitomycin (UGN-1016-9, Jelmyto [https://www.fda.gov/news-events/press-announcements/fda-approves-first-therapy-treatment-low-grade-upper-tract-urothelial-cancer]) that has been demonstrated to statistically significantly improve complete response after administration at the time of ablation of low-grade renal pelvis tumors (25). How this therapeutic development will impact the utilization of endoscopic management of low-risk UTUC on a large scale remains to be seen.

With respect to costs associated with UTUC diagnosis, we report statistically significantly higher overall costs associated with a diagnosis of high-risk UTUC when compared with low-risk disease. To date, no study has reported cost differences by risk strata; most reports focus on the direct costs of surgical procedures (ie, laparoscopic vs open surgical approaches to radical nephroureterectomy) (26,27). In our study, the costs incurred by high-risk patients were statistically significantly greater than those by low-risk patients. The one exception to this pattern was related to patients who underwent 2 endoscopic interventions, without ultimately progressing to nephroureterectomy. This may reflect an unmeasured characteristic of these patients who, even with high-risk disease, undergo iterative endoscopic surgeries and not nephroureterectomy (ie, presence of a costly comorbidity precluding surgical candidacy for extirpative procedure). Notably, although median costs at a year after diagnosis were higher in the total cohort among patients who underwent upfront nephroureterectomy as compared with those who underwent a single endoscopic intervention ($81 787 vs $72 463), if a patient was initially managed endoscopically and ultimately required radical nephroureterectomy, his or her total costs were higher than those who underwent a single surgical procedure.

Although we did not investigate granular cost contribution of each diagnostic study, procedure and/or surgery, the global annual cost following diagnosis is a reflection of care provided to a UTUC patient, in aggregate. In general, endoscopic management is typically a less expensive outpatient procedure compared with the hospitalization required after nephroureterectomy; it also is associated with fewer costly complications. However, one might hypothesize, that if iterative endoscopic procedures are performed, the financial burden of endoscopically managed patients may be higher with surveillance over time than those who undergo a single more expensive procedure (11). Also to be considered, with the removal of a renal unit as is the case with nephroureterectomy, there is increased risk of renal insufficiency and, in a minority of patients, progression to renal failure at a high cost (28). In an exploratory analysis, we found that only 2.9% (119 of 4114) of patients had a diagnosis of end-stage renal disease in the year prior to their UTUC diagnosis; this increased to 9.1% (373 of 4114) in the year after diagnosis and was higher among patients who underwent nephroureterectomy vs those who did not (10.2% vs 4.4%). Ten percent of patients is a nontrivial proportion, and managing subsequent sequelae of end-stage renal disease is expensive (29). A recent systematic review of the economic burden of treating UTUC highlighted the relative lack of data on the subject and the need for rigorous modeling of costs associated with managing low-risk disease, in particular, where treatment options are less constrained and have expanded to include the use of the previously mentioned novel chemo-ablative agent (20,25). The fact that, in our study, claims in the outpatient setting appear to drive the cost differential emphasizes the point that granular inspection of specific health needs—be they related to oncologic follow-up, management of comorbidities, or management of treatment sequelae—should be the next area of study.

Finally, we report both overall and cancer-specific survival, stratified by risk. Patients with high- vs low-risk disease had worse survival outcomes, even after controlling for patient and tumor characteristics and type of treatment. Although we did not perform a competing risks survival analysis, when comparing overall survival to cancer-specific survival, the proportion of noncancer-related deaths are appreciable, likely reflecting comorbid conditions and the advanced age of our patient population. As epidemiologic data have suggested a subtle but demonstrable increase in UTUC incidence in recent years, an increase in highly lethal advanced disease, and a relative stagnation of survival outcomes, these findings on the whole highlight the need for advancing effective and cost-contained management strategies (2,4,30).

This retrospective analysis is prone to selection bias and confounding. To handle missing data, we assessed outcomes based on unimputed vs imputed data, which resulted in similar conclusions using complete data, unimputed data, and imputed data. However, our use of multivariable analyses—adjusting for multiple demographic and clinical characteristics—helped address this limitation. Although billing claims data have been demonstrated to be robust capture of many events, we lacked the ability to determine certain patient, tumor, and treatment characteristics at a level of granularity that could have strengthened our analysis. For example, we were unable to determine tumor focality to include in our risk stratification; theoretically, this may have led to misclassification of a small number of patients as low risk and explain a proportional increase in nephroureterectomy use in the low-risk group. We could not distinguish between histology on biopsy and histology on surgical pathology from radical nephroureterectomy; the pathologic staging from the most definitive procedure is reported in SEER. We did not ascertain utilization of intracavitary instillation of either bacillus Calmette-Guerin or Mitomycin C at the time of endoscopic intervention, which is considered a component of the ideal kidney-sparing surgical approach. It is possible that the proportion of older patients with low-risk disease being managed endoscopically has increased in the past 7 years, and as more modern registry data become available, this should be explored. In addition, we recognize that Medicare is the federal insurance program for individuals 65 years and older and that cost data from a single payer may not be generalizable to younger populations with varying insurance carriers.

Despite these limitations, this comprehensive assessment of management, cost, and outcomes of all patients with nonmetastatic UTUC contributes to a greater understanding of the overall landscape of care provision. In the future, as new evidence emerges in the management of UTUC—timing of chemotherapy administration, potential roles for immunotherapy, utilization of intracavitary agents, advances in noninvasive diagnostics such as advanced imaging and circulating tumor DNA—it will be of the utmost importance to consider not only safety and efficacy of interventions but also their costs at a national level and for individual patients (31).

UTUC continues to be managed primarily with nephroureterectomy, regardless of risk stratification, with a minority undergoing renal preservation. Substantial direct health-care costs are associated with a diagnosis of low- and high-risk UTUC, with the latter being more costly and largely driven by outpatient costs. Patients with high-risk UTUC have worse survival. As necessary advances in effective perioperative treatments are brought to the fore, including neoadjuvant and adjuvant systemic therapy, novel delivery mechanisms of local therapy, and further refinement of kidney-sparing surgery, it will be critical to delineate associated costs to best provide patient-centered, value-based care.

## Funding

This study was conducted with the support of a Department of Defense Peer Reviewed Cancer Research Program (PRCRP) Career Development Award (W81XWH1710576) (SBW). VS is supported by the Veteran’s Affairs Health Services Research and Development Fellowship. KEF is supported by H&H Lee UCLA Surgical Scholars Program. KC receives research support from UroGen Pharma.

## Notes

**Role of the funders:** The funders had no role in the design of the study; the collection, analysis, and interpretation of the data; the writing of the manuscript; and the decision to submit the manuscript for publication.

**Disclosures:** The authors declare no potential conflicts of interest.

**Disclaimer:** The content is solely the responsibility of the authors and does not necessarily represent the official views of SEER-Medicare.

**Acknowledgements:** The content is solely the responsibility of the authors and does not necessarily represent the official views of the National Institutes of Health. This study used the linked Surveillance, Epidemiology, and End Results (SEER)–Medicare linked databases. The interpretation and reporting of these data are the sole responsibility of the authors. The authors acknowledge the efforts of the Applied Research Program, NCI; the Office of Research, Development, and Information, CMS; the Information Management Services (IMS), Inc; and the SEER program tumor registries in the creation of the SEER database. Katherine E. Fero, Yong Shan, Patrick M. Lec, Vidit Sharma, Aditya Srinivasan, Giri Movva, Jacques Baillargeon, Karim Chamie, Stephen B. Williams. This research project was funded by a research grant from UroGen Pharma (2020-0776).

**Author contributions:** Conceptualization- Katherine E. Fero, Yong Shan, Karim Chamie, Stephen B. Williams. Data curation- Stephen B. Williams, Yong Shan. Formal analysis- Yong Shan. Funding acquisition- Karim Chamie, Stephen B. Williams. Investigation- Katherine E. Fero, Yong Shan, Patrick M. Lec, Vidit Sharma, Aditya Srinivasan, Giri Movva, Jacques Baillargeon, Karim Chamie, Stephen B. Williams. Methodology- Katherine E. Fero, Yong Shan, Patrick M. Lec, Vidit Sharma, Aditya Srinivasan, Giri Movva, Jacques Baillargeon, Karim Chamie, Stephen B. Williams. Project administration- Stephen B. Williams. Resources- Stephen B. Williams. Software- Yong Shan, Stephen B. Williams. Supervision- Karim Chamie, Stephen B. Williams. Validation- Katherine E. Fero, Yong Shan, Patrick M. Lec, Vidit Sharma, Aditya Srinivasan, Giri Movva, Jacques Baillargeon, Karim Chamie, Stephen B. Williams. Visualization- Katherine E. Fero, Yong Shan, Patrick M. Lec, Vidit Sharma, Aditya Srinivasan, Giri Movva, Jacques Baillargeon, Karim Chamie, Stephen B. Williams. Writing—original draft- Katherine E. Fero, Yong Shan, Patrick M. Lec, Vidit Sharma, Aditya Srinivasan, Giri Movva, Jacques Baillargeon, Karim Chamie, Stephen B. Williams. Writing—review & editing- Katherine E. Fero, Yong Shan, Patrick M. Lec, Vidit Sharma, Aditya Srinivasan, Giri Movva, Jacques Baillargeon, Karim Chamie, Stephen B. Williams.

## Data Availability

This study used the SEER-Medicare database. The interpretation and reporting of these data are the sole responsibility of the authors. The data underlying this article were provided by SEER-Medicare under license/by permission. Data will be shared on request to the corresponding author with permission of SEER-Medicare.

## Supplementary Material

pkab085_Supplementary_Data
