# Supplementary material for: Treatment Patterns, Outcomes, and Costs Associated With Localized Upper Tract Urothelial Carcinoma
Source: JNCI Cancer Spectr. 2021 Oct 1;5(6):pkab085. doi: 10.1093/jncics/pkab085 (PMC8599752; doi:10.1093/jncics/pkab085)

SUPPLEMENTARY MATERIAL

Supplementary Table 1. Current Procedural Terminology (CPT), International Classification of Diseases (ICD), and Healthcare Common Procedure Coding System (HCPCS) codes used to classify treatment.

Treatment or Diagnosis	Description	CPT	ICD	HCPCS J-Code
Laparoscopic/open surgery	–	50220, 50225, 50230, 50234, 50236, 50240, 50543, 50545, 50546, 50548, 50549, 50550, 50660, 50947, 50948, 50949	–	–
Laparoscopic/open surgery	–	-	55.x, 56.4	–
Endoscopy	renal endoscopy [with or without intervention]	50551, 50553, 50555, 50557, 50559, 50561, 50562, 50570, 50572, 50574, 50575, 50576, 50578, 50580	–	–
Endoscopy	ureteral endoscopy [with or without intervention]	50951, 50953, 50955, 50957, 50959, 50961, 50970, 50972, 50974, 50976, 50980, 52335-9, 52344, 52345, 52346, 52351-5	–	–
Systemic Therapy	Methotrexate	–	–	J9250, J9260
Systemic Therapy	cisplatin			J9060, J9062
Systemic Therapy	carboplatin	–	–	J9045
Systemic Therapy	vinblastane	–	–	J9360
Systemic Therapy	doxorubicin	–	–	J9000, J9010, J9001
Systemic Therapy	cyclophosphamide	–	–	J9070, J9080, J9091-8
Systemic Therapy	gemcitabine	–	–	J9201
Systemic Therapy	paclitaxel	–	–	J9265

Systemic Therapy	docetaxel	–	–	J9170
Systemic Therapy	ifosfamide	–	–	J9208
End-stage Renal Disease	End-stage renal disease	–	585.6, 586.x	–

Supplementary Table 2. Full results of Cox proportional Hazard Survival analysis.

Groups	Overall Survival HR (95% CI)	Cancer Specific Survival HR (95% CI)
Risk group		
Low	1.00 (Reference)	1.00 (Reference)
High	1.78 (1.62 to 1.96)	4.05 (3.11 to 5.27)
Sex		
Male	1.00 (Reference)	1.00 (Reference)
Female	0.91 (0.83 to 1.00)	1.07 (0.91 to 1.27)
Age at diagnosis, y		
66-70	1.00 (Reference)	1.00 (Reference)
71-75	1.22 (1.04 to 1.43)	0.83 (0.63 to 1.10)
76-80	1.54 (1.32 to 1.80)	1.01 (0.77 to 1.32)
>80	2.28 (1.97 to 2.63)	1.64 (1.29 to 2.09)
Race		
Black	0.96 (0.75 to 1.22)	0.82 (0.53 to 1.28)
Hispanic	0.97 (0.69 to 1.36)	0.96 (0.51 to 1.82)
White	1.00 (Reference)	1.00 (Reference)
Other	0.88 (0.73 to 1.06)	0.88 (0.62 to 1.24)
Marital status		
Single	1.00 (Reference)	1.00 (Reference)
Married	0.84 (0.74 to 0.96)	0.95 (0.74 to 1.22)
Unknown	0.97 (0.84 to 1.12)	0.96 (0.73 to 1.26)
Census Region		
West	1.00 (Reference)	1.00 (Reference)
Midwest	0.92 (0.82 to 1.03)	0.74 (0.59 to 0.91)
South	1.03 (0.89 to 1.19)	1.22 (0.96 to 1.54)
Northeast	1.14 (1.02 to 1.28)	0.92 (0.74 to 1.14)
Median Income		
Bottom Quartile	1.00 (Reference)	1.00 (Reference)
Second Quartile	1.06 (0.94 to 1.19)	1.11 (0.90 to 1.37)
Third Quartile	1.00 (0.89 to 1.14)	1.05 (0.84 to 1.32)
Fourth Quartile	0.99 (0.87 to 1.13)	0.89 (0.70 to 1.14)
Comorbidity, No.		
0	1.00 (Reference)	1.00 (Reference)
1	1.31 (1.18 to 1.45)	1.27 (1.04 to 1.54)
2	1.38 (1.21 to 1.56)	1.49 (1.18 to 1.88)

≥3	2.10 (1.87 to 2.37)	1.81 (1.46 to 2.26)
Year of diagnosis		
2004	1.00 (Reference)	1.00 (Reference)
2005	0.86 (0.73 to 1.00)	0.83 (0.61 to 1.12)
2006	0.91 (0.77 to 1.08)	0.76 (0.55 to 1.03)
2007	0.93 (0.79 to 1.10)	0.83 (0.61 to 1.13)
2008	0.95 (0.81 to 1.12)	0.76 (0.55 to 1.03)
2009	0.89 (0.75 to 1.06)	0.76 (0.56 to 1.03)
2010	0.85 (0.71 to 1.02)	0.93 (0.68 to 1.26)
2011	0.76 (0.62 to 0.93)	0.82 (0.59 to 1.14)
2012	0.96 (0.79 to 1.18)	0.67 (0.46 to 0.96)
2013	0.82 (0.65 to 1.05)	0.71 (0.48 to 1.07)
Definitive surgical intervention		
Laparoscopic or open surgery	1.00 (Reference)	1.00 (Reference)
Endoscopic only	1.45 (1.25 to 1.69)	1.34 (1.02 to 1.76)
None	1.60 (1.31 to 1.96)	1.68 (1.19 to 2.37)
Lymph node dissection		
No	1.00 (Reference)	1.00 (Reference)
Yes	1.22 (1.09 to 1.37)	1.48 (1.23 to 1.77)

[a] CI = confidence interval; HR = hazard ratio

Supplementary Figure 1. Cumulative monthly costs of care in the year after diagnosis, stratified by risk classification.

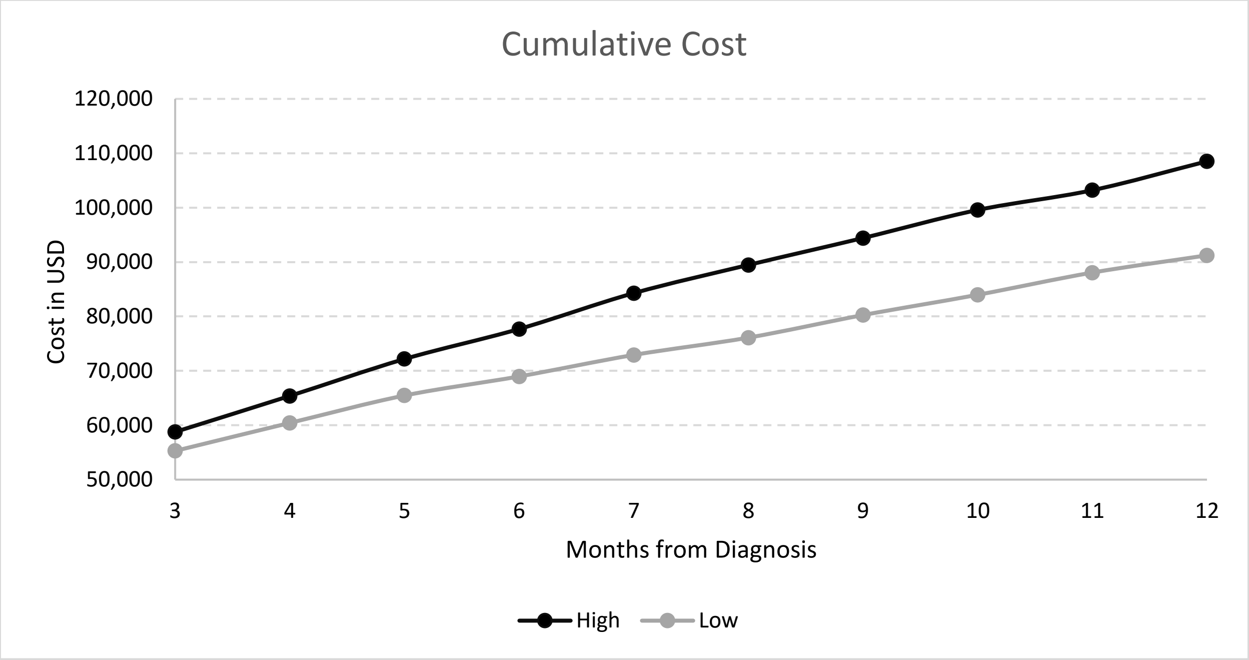

Supplement: pkab085_Supplementary_Data [file pkab085_supplementary_data.pdf]
